# Sociodemographic, lifestyle and clinical factors associated with good performance in paired associates learning (PAL) test in patients with schizophrenia

**DOI:** 10.1192/j.eurpsy.2021.1450

**Published:** 2021-08-13

**Authors:** M. Taivalantti, M. Kerkelä, A.-H. Halt, J. Barnett, J. Veijola

**Affiliations:** 1 Department Of Psychiatry, University of Oulu, Oulu, Finland; 2 Department Of Psychiatry, Cambridge Cognition, UK, University of Cambridge, UK, Cambridge, United Kingdom

**Keywords:** schizophrénia, visual memory and new learning

## Abstract

**Introduction:**

Memory and learning deficits are central among cognitive deficits in schizophrenia. However, to a varying proportion ca. 20-25% of patients could not be considered deficit.

**Objectives:**

Description of sociodemographic, lifestyle and clinical factors related to good performance in PAL-test in schizophrenia patients.

**Methods:**

Participants (N=4500) were members of the Finnish SUPER study on the genetic mechanisms of psychotic disorders (SUPER). The database of the Northern Finland Birth Cohort 1966 (NFBC 1966) was utilized as a reference data. Visual memory and new learning were assessed using Cambridge Neuropsychological Test Automated Battery (CANTAB) Paired Associates Learning (PAL) test. The 50^th^ percentile scores (10 error score or less) for outcome measure total errors adjusted (TEA) of NFBC 1966 was used as a cut-off for good performance in PAL test.

**Results:**

The sociodemographic and lifestyle factors related good performance for both sexes were: younger age (p<.001), higher basic education (p <.001), independent form of dwelling (p<.001), hazardous drinking (p <.001), cannabis use (p <.001) and being married (females p = 0.009, males p = 0.049). The clinical factors related to good performance for both sexes were not using antipsychotic medication regularly (p <.001), not using all psychotropic medication (females p=0.05, males p <.001), less hospitalization times due to psychosis (p <.001), younger age at first hospitalization due to psychosis (p <.001), lower number of hospitalization days (p <.001) and lower percentage of time in hospital after first psychosis episode (p <.001).

**Conclusions:**

Several factors related to good performance in the PAL–test in the crude analysis without any confounders.

